# Viburnum Prunifolium

**Published:** 1881-12-20

**Authors:** A. G. Smythe

**Affiliations:** Mississippi


### VIBURNUM PRUNIFOLIUM.

BY A. G. SMYTHE, M. D., OF MISSISSIPPI.

For a number of years, the current medical literature has been teeming with glowing accounts, and each succeeding contributor doing his utmost to excel his predecessor, in sounding the virtues, claims and praises of Viburnum Prunifolium, in the prevention and arrest of abortion, miscarriage and all the concomitant troubles attending that condition. Notwithstanding such an array of witnesses, many of whom I well know and esteem highly, personally and professionally; it is with no factious spirit of fault-finding or wish to set up an independent opinion, but from a fair trial and an unbiassed observation, that I have been driven to a different conclusion.

I set out upon a trial of the remedy with flattering hopes and fond expectations, from the many praises of its virtues which I had heard. I was led to believe that I would find in it an anchor which could be relied upon in the most dreadful storm, and provided the craft had not sprung too large a leak or parted her timbers in the middle, would ride safely through; all that would be necessary would be time to repair the injuries incident to such a gale, the craft could be kept at sea, finish the voyage in proper time, arrive safely in the port of destination and deliver the cargo in good order and condition, and in due time be ready for sea again. But, alas for human hopes! Mine were doomed to meet the most painful and signal disappointment. With all the remedies, old or new, indigenous or exotic, in those cases, I have not met with such complete disappointment. With no other remedy or treatment has such uniform failure occurred in a practice of more than forty-five years, very largely obstetric.

I will not consume your space or the time of your readers, by offering an array of cases in due form. A fair trial, in a variety of cases, for eight years, with a total failure in each, when chifly relied upon, would cause the most stubborn advocate to abandon it in disappointment, mortification, disgust and disgrace, having lost professional character more than once from relying upon it. Let it not be supposed for a moment that I am opposed to the introduction of new remedies—the reverse is what I am charged with. I ask pardon for disagreeing with a number of my brethren, who think well of the remedy in question. I candidly think they have been deceived; the benefits attributed to it have been, at best, only apparent or accidental; and the credit due to some other remedy, or to the powers of repair, reproduction or spontaneous restoration.

If your readers will bear with me one moment further, I will just say: much of the hasty zeal of many of our well-meaning brothers in the good cause, retard rather than advance the cause of new remedies in therapeutics, by hurrying crude and imperfect trials, partial experiments and loose observations into the pages of the medical literature of the period.

